# Experience with hepatectomy in a patient with focal nodular hyperplasia combining with constitutional indocyanine green excretory defect: a case report

**DOI:** 10.3389/fmed.2026.1675917

**Published:** 2026-01-22

**Authors:** Tengfei Wang, Yunyi Li, Xiaodong Sun, Guoyue Lv

**Affiliations:** Department of Hepatobiliary and Pancreatic Surgery, General Surgery Center, The First Hospital of Jilin University, Changchun, China

**Keywords:** constitutional indocyanine green excretory defect, focal nodular hyperplasia, indocyanine green retention rate at 15 min, hepatectomy, case report

## Abstract

This report details the first documented instance of successful hepatic resection performed in a patient presenting with focal nodular hyperplasia (FNH) concomitant with constitutional indocyanine green (ICG) excretory defect – an exceptionally rare hepatic transport disorder initially characterized in 1974. A male patient in his early 20s was diagnosed with FNH necessitating surgical evaluation. Preoperative assessment revealed a profoundly elevated ICG retention rate at 15 min (ICG-R15) of 66.7%, indicating severely impaired clearance based on conventional interpretation. Crucially, however, comprehensive evaluation demonstrated discordantly normal standard liver function biochemical parameters (including bilirubin, transaminases, albumin, coagulation profile) and entirely unremarkable histopathological findings obtained via percutaneous biopsy of radiologically normal liver parenchyma. This definitive constellation of findings confirmed the diagnosis of constitutional ICG excretory defect, effectively excluding intrinsic hepatic parenchymal dysfunction or significant functional impairment. Consequently, proceeding with hepatic mass resection was deemed justified. The surgical intervention and immediate postoperative course were entirely uneventful, characterized by hemodynamic stability, absence of biochemical liver failure, and no complications during the critical recovery phase, with histopathology confirming FNH. This case constitutes a seminal demonstration that ICG clearance kinetics are inherently unreliable and potentially misleading as a sole indicator of functional hepatic reserve in patients harboring this specific excretory defect who are candidates for hepatectomy. Our findings establish the critical principle that the imperative for a multifaceted preoperative evaluation strategy that transcends reliance on ICG kinetics alone to safely guide surgical intervention in this unique patient population.

## 1 Introduction

The indocyanine green (ICG) clearance test is now widely used in the preoperative assessment of hepatic resection and is a useful method for assessing liver functional reserve. However, in patients with constitutional indocyanine green excretory defect, it is difficult for this test to accurately assess liver functional reserve to guide hepatic resection. We present a case of a young male patient with focal nodular hyperplasia (FNH) and constitutional indocyanine green excretory defect, in whom standard ICG R15 values were abnormally elevated (66.7% and 53.3%) despite normal liver biochemistry and Child-Pugh Grade A status. The patient doesn’t exhibit abnormal symptoms or signs. Further evaluation with Gd-EOB-DTPA-enhanced MRI and liver biopsy confirmed FNH without underlying liver disease. The patient successfully underwent hepatic resection, with postoperative pathology confirming the diagnosis. For patients with constitutional indocyanine green excretory defect, our experience emphasizes the necessity of developing alternative strategies for assessing liver reserve function to ensure the rationality of surgery, as well as the potential safety risks.

## 2 Case description

A male patient in his early 20 s with a 1-month history of focal liver lesions was admitted to the hospital. The patient did not present with any symptoms of nausea, vomiting, abdominal pain, jaundice, or a history of hepatitis. The preoperative levels of aspartate aminotransferase (AST), alanine aminotransferase (ALT), total bilirubin (TBIL), direct bilirubin (DBIL), and alpha-fetoprotein (AFP) were within the normal range. An enhanced magnetic resonance imaging (MRI) of the liver at an external medical facility revealed the presence of a space-occupying lesion in the upper right posterior lobe of the liver, with an estimated volume of 4.6 cm × 5.9 cm × 6.1 cm. The possibility of focal nodular hyperplasia was considered in the differential diagnosis. The Child-Pugh score was 5 points, which indicated a grade of A. The patient presented with an ALBI score of −2.67 and a MELD score of 6, along with a normal platelet count and an absence of portal hypertension. The patient underwent an ICG test, and the ICG retention rate at 15 min (ICG R15) was 66.7%. The test was repeated in the afternoon of the same day, and ICG R15 was 53.3%.

**FIGURE 1:**
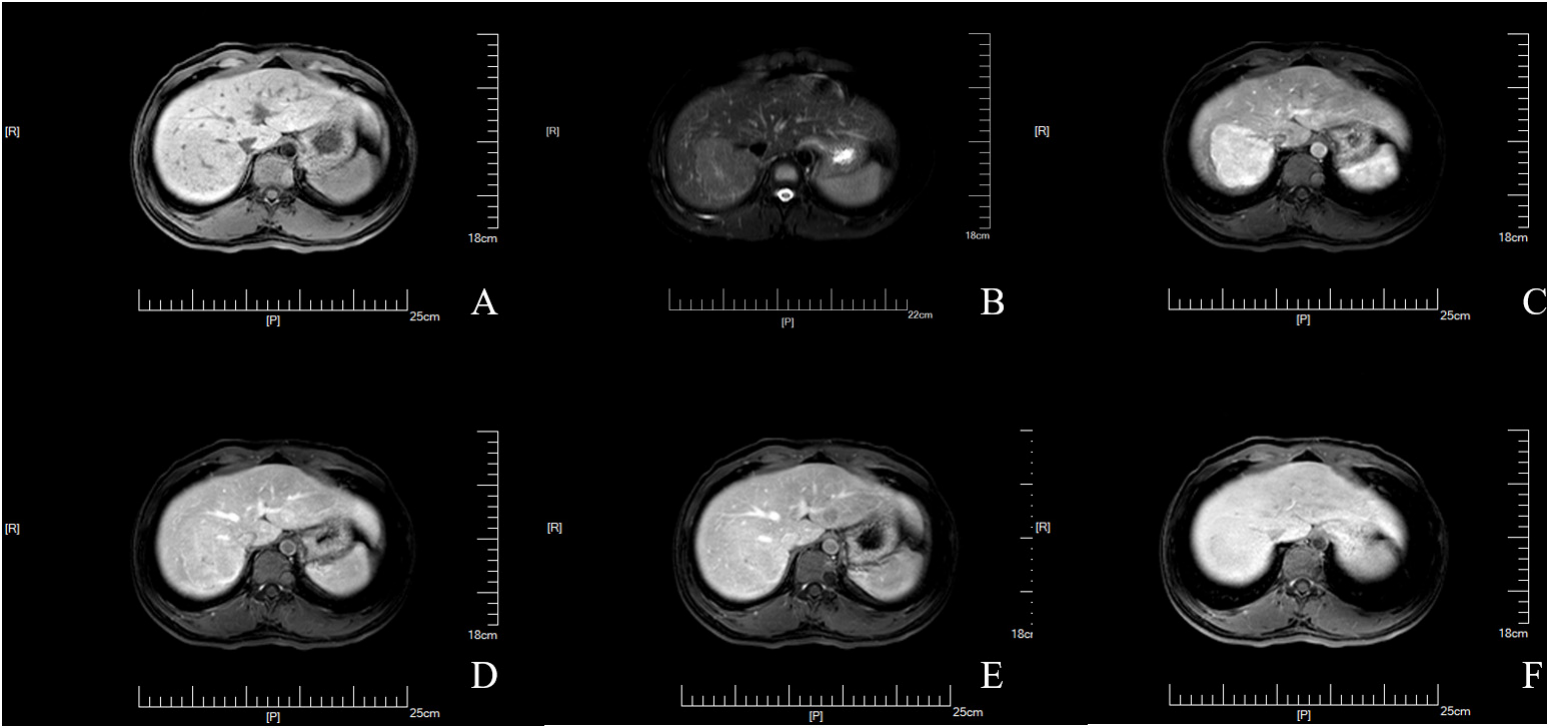
The lesion demonstrates typical imaging characteristics of FNH on Gd-EOB-DTPA-MRI. **(A)** T1-weighted image. **(B)** T2-weighted image. **(C)** Arterial phase. **(D)** Portal venous phase. **(E)** Transitional phase. **(F)** Hepatobiliary phase.

**FIGURE 2:**
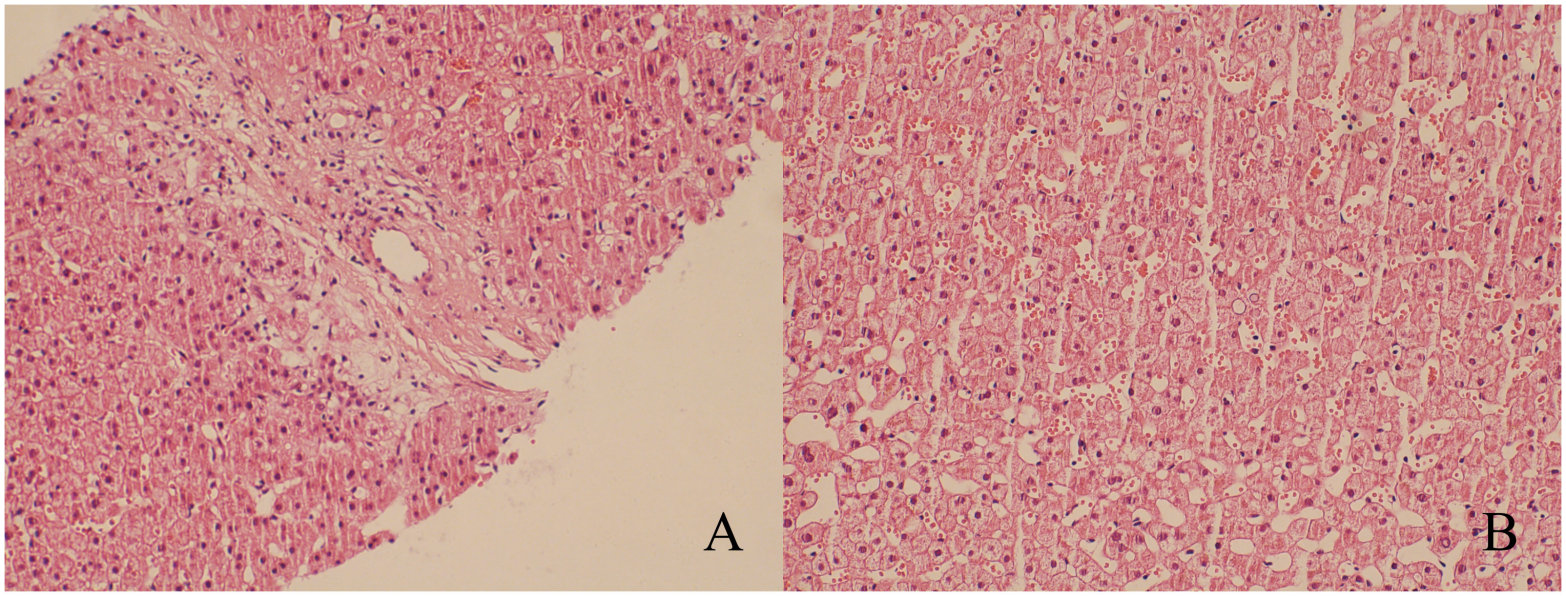
Results of puncture biopsy (hematoxylin-eosin staining, ×200). **(A)** The diseased tissue exhibited edematous degenerated banded fibrous tissue and small bile duct hyperplasia, which led to the diagnosis of FNH. **(B)** The liver tissue exhibited no discernible abnormalities.

**FIGURE 3:**
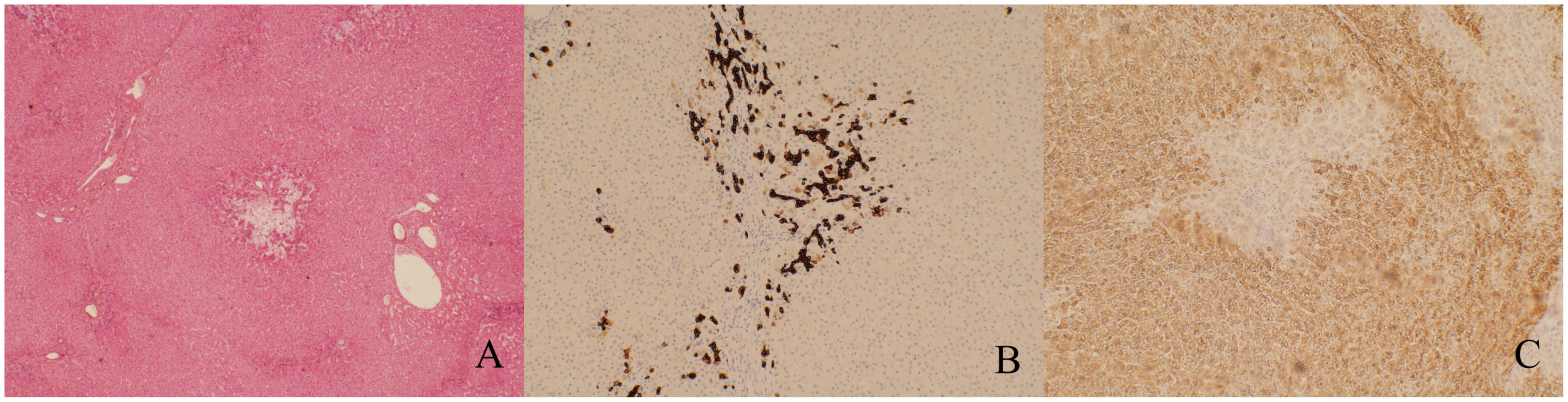
Postoperative pathological and immunohistochemical findings. **(A)** The lesion tissue displays the presence of scar fibers and abnormally thick-walled vessels (hematoxylin-eosin staining, ×40). **(B)** The cells of ductular reaction are positive for cytokeratin 7 (CK7) (immunohistochemical staining, ×100). **(C)** Immunohistochemical staining for glutamine synthetase (GS) demonstrated a map-like appearance (immunohistochemical staining, ×100).

To further exclude primary liver disease, the patient underwent a gadolinium ethoxybenzyl diethylenetriamine pentaacetic acid (Gd-EOB-DTPA)-enhanced MRI, which suggested the presence of a space-occupying lesion in the upper right posterior lobe of the liver. This was considered to be FNH (Figure 1), with no other abnormalities observed in the liver background. A puncture biopsy of the lesion and the surrounding normal liver tissue was also performed, and the biopsy pathology indicated that the lesion was FNH (Figure 2A), with no evidence of abnormality in the normal liver tissue (Figure 2B). The patient was thus diagnosed with FNH with constitutional indocyanine green excretory defect. Although the lesion is considered benign, its large size presents long-term uncertainties, including risks of continued growth and rupture. We considered treatment options such as conservative observation and transarterial embolization. However, given the patient’s young age and generally good health, both the patient and their family preferred radical surgical treatment. Transarterial embolization is typically reserved for patients with surgical contraindications, whereas this patient had no underlying conditions and demonstrated good actual liver function. Surgical resection would not only provide a definitive diagnosis but also achieve curative intent. After comprehensive risk-benefit analysis, considering the patient’s favorable baseline condition, low surgical risk, and the avoidance of long-term follow-up uncertainties, surgery was ultimately deemed the most appropriate choice. Subsequently, the patient underwent radical surgical treatment via open hepatectomy, with an incision made along the right subcostal margin extending to the midline. Intraoperative exploration revealed a smooth liver surface with well-proportioned lobes. A firm mass was palpable in the right hepatic lobe, consistent with preoperative imaging findings. Ultrasound was employed to delineate tumor margins and assess its relationship with major vessels. Hepatic resection was performed along the tumor margin, excising the tumor along with 1 cm of surrounding liver tissue. Postoperative pathology confirmed the diagnosis of FNH (Figure 3) with negative surgical margins. Immunohistochemistry was then performed. The immunohistochemical analysis yielded the following results: CK7 (bile ducts +), Ki-67 (+1%), GPC-3 (−), CD34 (partially +), GS (map-like +), β-catenin (membranes +), and a specific stain: reticulin staining (hepatic plate disruption −).

The patient recovered well postoperatively and was discharged on the sixth postoperative day. According to the International Liver Surgery Study Group definition, no postoperative hepatic failure occurred. Additionally, no complications of grade ≥ II were observed based on the Clavien–Dindo classification system. The patient and their family are highly satisfied with the effectiveness of the treatment. An ICG test was conducted on the fifth postoperative day, and the ICG 15-min retention rate was 65.7%. Genome-wide testing was performed on the patient, but no clear causative gene was identified.

All patient details have been anonymized to ensure patient privacy, and written informed consent was obtained from the patient prior to treatment. Because all patient information has been de-identified, written informed consent for publication was not required. In addition, because of the nature of this study (case report), formal ethics committee approval was not required. The reporting of this study conforms to CARE guidelines (1).

## 3 Discussion

Constitutional indocyanine green excretory defect is an extremely rare disease, predominantly observed in Japan. Furthermore, the number of cases of hepatectomy in patients with constitutional indocyanine green excretory defect is exceedingly scarce, with only nine reported cases (Table 1) (2–8). Notably, none of these patients suffered from FNH. In this paper, we present the inaugural case of hepatectomy in a patient with FNH concomitant with constitutional indocyanine green excretory defect. Patients with constitutional indocyanine green excretory defect do not present with any clinical symptoms, and thus, diagnosis is only possible through the identification of abnormal ICG clearance test results (9).

**TABLE 1:** Previously reported cases of constitutional indocyanine green excretory defect undergoing hepatectomy.

Number	References	Age/sex	Disease	ICG R15	Child-Pugh grade	Operation	Postoperative complications
1	(2)	78/F	Bile duct cystadenocarcinoma	79.30	A	Left lateral sectionectomy	None
2	(3)	77/M	HCC	77.10	B	Left medial sectionectomy + resection of the ventral region of the anterior segment	Hyperbilirubinemia
3	(4)	81/M	HCC	79.10	A	Central hepatectomy	None
4	(5)	83/M	HCC	76.20	A	Partial hepatectomy (S4)	None
5	(6)	58/M	HCC	83.00	A	Partial hepatectomy (S5 + S8)	None
6	(6)	70/M	Liver metastases from colon cancer	68.00	A	Partial hepatectomy (S8)	None
7	(6)	69/M	Hilar cholangiocarcinoma	73.00	A	Choledochectomy and extended medial segmentectomy of the liver	None
8	(7)	68/M	HCC	84.90%	A	Partial hepatectomy (S8)	None
9	(8)	64/M	HCC	70%	A	Partial hepatectomy (S5)	None

F, female; M, male; ICG R15, indocyanine green retention rate at 15 min; HCC, hepatocellular carcinoma; ND, not described.

ICG is a tricarbocyanine dye that is primarily taken up by hepatocytes and excreted through the biliary system. Abnormalities in hepatic blood flow, impaired biliary excretion, and impaired hepatocellular function have been demonstrated to affect ICG clearance (3, 5, 10–13). In liver surgery, ICG is often used for preoperative evaluation of liver surgery, intraoperative fluorescence imaging, and postoperative prediction of liver failure. The ICG clearance test is regarded as a valuable tool for preoperative assessment of hepatic functional reserve, with a growing presence in clinical practice. ICG R15 is commonly used as one of the indicators to assess liver reserve function. Some research centers have established criteria to assess the safety limits of hepatectomy based on parameters such as ICG R15. The University of Zurich combined the pathological state of the liver parenchyma, Child-Pugh score, portal hypertension, ICG R15, and other parameters to deduce the status of patients’ liver reserve function and the corresponding safety limit of hepatectomy (14). The University of Tokyo, Japan, established a decision tree to evaluate the safety limit of hepatectomy mainly based on abdominal fluid, bilirubin level, and ICG R15 (15).

However, in patients with constitutional indocyanine green excretory defect, abnormally elevated ICG R15 results in the loss of reliability in assessing the extent of liver injury and hepatic reserve function. Clinicians are unable to assess whether surgery is contraindicated and the maximum tolerable hepatic resection volume by the indocyanine green clearance test. Consequently, a more comprehensive preoperative liver assessment is required in such patients. Kawamura et al. (11) reported the results of a study on the assessment of hepatic functional reserve using a transformed ICG R15 based on the calculation of 99mTc-GSA scintigraphy. Recently, several studies have indicated Gd-EOB-DTPA-enhanced MRI as a potential method to assess liver reserve function (16–18). Except for abnormal results in the ICG clearance test, this case showed no significant abnormalities in general liver function biochemical markers, platelet count, Child-Pugh score, ALBI score, MELD score, or liver biopsy. Additionally, the Gd-EOB-DTPA-enhanced MRI did not identify any underlying liver disease in the patient. Consequently, despite the ICG R15 exceeding 50%, it was determined that the patient’s hepatic reserve was sufficiently functional to permit surgical treatment. This article proposes that assessment of liver reserve function and the extent of liver injury in patients with constitutional indocyanine green excretory defect needs to be accomplished by a combination of general liver function tests, imaging, and pathologic histology.

*In vitro* studies have demonstrated that ICG is predominantly taken up by hepatocytes via organic anion transporting polypeptide 1B3 (OATP1B3, gene symbol SLCO1B3) and sodium-taurocholate cotransporting polypeptide (NTCP, gene symbol SLC10A1) (10). In healthy livers, ICG is transported via the bloodstream to hepatic sinusoids, where it is primarily actively taken up into hepatocytes by OATP1B3 on the basal membrane and ultimately excreted into bile (10). As the primary and high-affinity transporter mediating ICG uptake in the liver, OATP1B3 deficiency impairs ICG uptake, leading to its accumulation in the bloodstream and resulting in markedly elevated ICG-R15 levels (4). However, the intrinsic functions of hepatocytes (such as synthesis and metabolism) and bile excretion functions are typically intact. This explains why patients’ conventional liver function markers (bilirubin, transaminases, coagulation function) and liver histopathology appear completely normal. Recent studies have implicated loss of OATP1B3 expression as the etiology of constitutional indocyanine green excretory defect and reported that homologous insertion of a 6.5 kbp long interspersed element (LINE-1, L1) retrotransposon into intron 5 of SLCO1B3 results in loss of its expression (19, 20). OATP1B3 exhibits distinctive transport properties. It has been observed that cholecystokinin octapeptide (CCK-8) is transported by OATP1B3 in the human liver (21). Furthermore, OATP1B3 is the sole hepatic organic anion-transporting polypeptide (OATP) that facilitates the transport of digoxin, docetaxel, and paclitaxel (22–24). The OATP1B3 protein is expressed at significant levels in certain gastrointestinal tumors, which represents a pivotal molecule in the delivery of methotrexate (MTX) to gastrointestinal tumors and its presence determines the sensitivity of tumor cells to MTX (25). Therefore, patients with constitutional indocyanine green excretory defect may be affected by OATP1B3 deficiency, which may affect the metabolism and targets of the relevant drugs and result in drug toxicity. Furthermore, the clearance of ICG from the bloodstream is analogous to the clearance of various endogenous and exogenous substances, including bilirubin, hormones, and certain drugs that share partially overlapping transporter carriers (10, 12). Patients with constitutional indocyanine green excretory defect may exhibit abnormal metabolism of related substances due to defects in the function of these common carriers.

Some studies suggest that the uptake of gadolinium ethoxybenzyl diethylenetriamine pentaacetic acid in liver parenchyma is associated with OATP1B3, and there is a correlation between OATP1B3 expression and the enhancement of liver parenchyma during the hepatobiliary phase (HBP) of EOB-MRI (26–28). It has been demonstrated that the absence of OATP1B3 expression limits the detectability of lesions during the hepatobiliary phase of EOB-MRI (6). In our case, Gd-EOB-DTPA-MRI demonstrated a homogeneous hepatic background without cirrhotic morphology. This radiologically excludes potential diffuse liver disease, providing further critical evidence supporting the patient’s well-preserved actual hepatic reserve function. Although EOB-MRI accurately detected the lesion in this case, we should integrate other imaging modalities when evaluating such patients to minimize the risk of lesion misdiagnosis. However, in the case we reported, the results of genetic testing of the patient did not suggest alterations in the OATP1B3-related genes. Thus, the etiology of constitutional indocyanine green excretory defect requires more detailed studies in the future.

The key insight from this case is that when ICG clearance significantly deviates from the patient’s overall clinical condition, clinical decisions should not rely solely on a single ICG kinetic parameter. We recommend a stepwise approach: repeat ICG testing to rule out technical errors, systematically evaluate liver function using ALBI, MELD scores, and platelet counts, and supplement assessments with functional imaging modalities such as Gd-EOB-DTPA-enhanced MRI when necessary. Multidimensional evaluation is critical to ensuring safe surgical intervention for these patients.

## 4 Conclusion

In conclusion, the hepatic reserve function of patients with constitutional indocyanine green excretory defect cannot be evaluated solely based on the traditional ICG clearance test. Instead, a comprehensive preoperative assessment of these patients is essential. Given the potential lack of transporter carriers in constitutional indocyanine green excretory defect, it is crucial to exercise caution when administering drugs to such patients.

## Data Availability

The original contributions presented in this study are included in this article/supplementary material, further inquiries can be directed to the corresponding authors.
